# Supplementary material for: Analysis of chi angle distributions in free amino acids via multiplet fitting of proton scalar couplings
Source: Magn Reson (Gott). 2024 Aug 19;5(2):103–20. doi: 10.5194/mr-5-103-2024 (PMC11570886; doi:10.5194/mr-5-103-2024)
Supplement: The supplement related to this article is available online at: https://doi.org/10.5194/mr-5-103-2024-supplement. [file mr-5-103-2024-supplement.zip › Syed2024/data/fit1d_fitnmr_output.tar.gz!/fit1d/thr/500/bmse000049_simulation_1_nmredata/1H/sparse_1d.pdf]

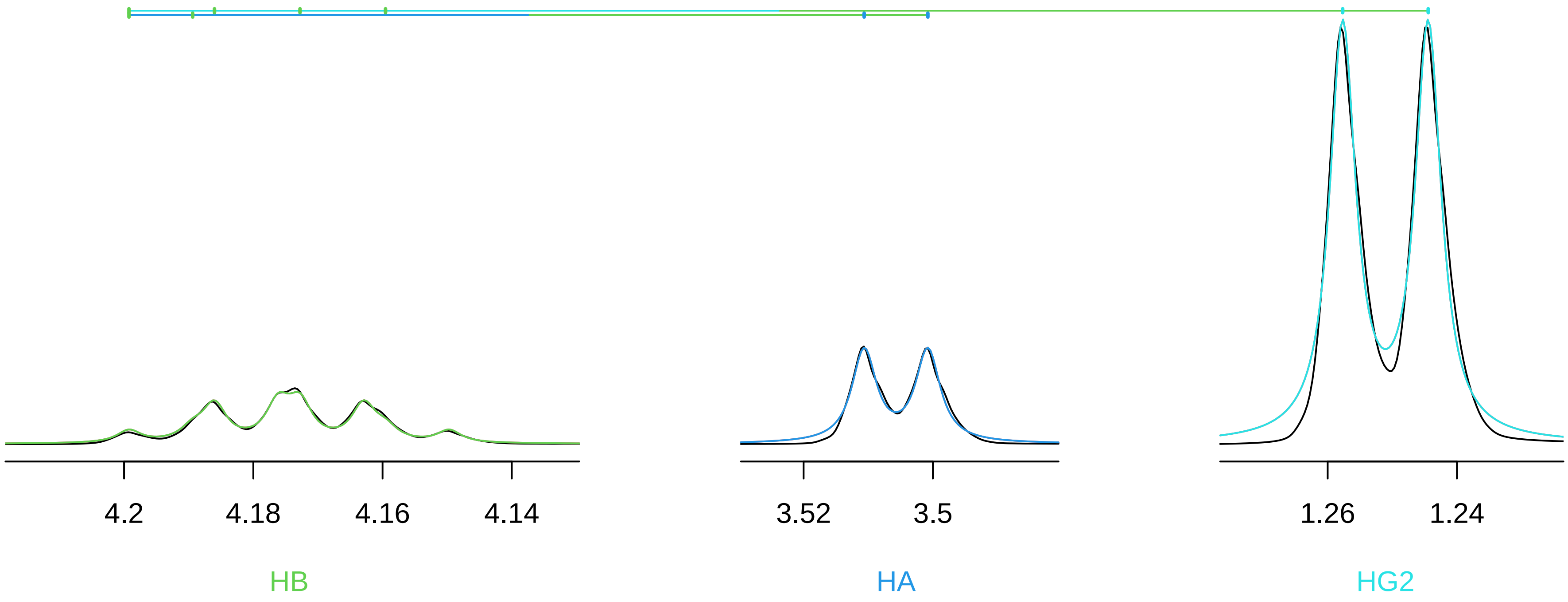

4.2
4.18
4.16
4.14
3.52
3.5
1.26
1.24
HB
HA
HG2
